# Perceived organizational support and kindergarten teachers’ voice behavior: the mediating role of psychological capital and the moderating role of establishment status

**DOI:** 10.3389/fpsyg.2025.1660484

**Published:** 2025-10-23

**Authors:** Yu Gou, Gang Zeng, Ying Xu, Xiao Xie

**Affiliations:** ^1^Chongqing Youth Vocational & Technical College, Chongqing, China; ^2^Faculty of Education, Khon Kaen University, Khon Kaen, Thailand; ^3^Chongqing Three Gorges Medical College, Chongqing, China; ^4^Chongqing University of Education, Chongqing, China

**Keywords:** kindergarten teachers, perceived organizational support (POS), voice behavior(VB), psychological capital (PsyCap), establishment status (ES)

## Abstract

Perceived organizational support is associated with employee voice behavior; however, existing research has paid little attention to the interplay between organizational and individual factors and the mechanisms through which they influence employee voice behavior. This study employed a questionnaire survey method, collecting data from 314 preschool teachers. The collected data were analyzed using Amos 27.0 and SPSS 27.0 statistical software. Additionally, the moderating effect of establishment status on the relationship between teachers’ perceived organizational support and psychological capital was not significant, providing theoretical insights for future research on kindergarten teachers.

## 1 Introduction

Employee voice behavior, as an organizational practice conducive to achieving mutual benefits for both employees and organizations, has gradually drawn attention in the field of education since the concept was first proposed by Hirschman, a distinguished scholar of the American Economic Association, in 1970. Teacher voice behavior refers to the proactive expression of constructive suggestions or opinions by educators to their organizations (Liu et al., 2016). Thus, it is not merely an internal communication practice within educational institutions but also a vital force for enhancing educational quality, improving student performance, boosting the profession’s appeal, and advancing educational democratization and social equity (UNESCO, 2016). However, despite its recognized value, teachers often encounter challenges when engaging in voice behavior. Factors such as hierarchical school structures (Pfister and Paljevic, 2024), fear of negative evaluations, and lack of leadership receptivity (Santagata et al., 2023) may hinder the emergence of teacher voice behavior. These challenges underscore the importance of exploring antecedents that encourage teacher voice behavior. Among various influencing factors, teachers’ perceived organizational support is considered a crucial predictor—specifically, their perception of whether the organization provides sufficient recognition, support, and security (Chen et al., 2022; Yan et al., 2025). Therefore, to foster a positive discourse environment and provide a valuable pathway for kindergarten teachers to better participate in kindergarten decision-making, further exploration of how kindergarten teachers’ perceived organizational support operates is necessary.

According to social exchange theory, perceived organizational support refers to an individual’s overall belief that “the organization values their contributions and cares about their well-being” (Eisenberger et al., 1986). When members experience higher levels of organizational support, they develop stronger feelings of belonging and loyalty, thereby becoming more willing to contribute their wisdom and efforts to the organization’s development (Desimone, 2009; Kim and Qu, 2020), ultimately enhancing work efficiency (Ling et al., 2006). Empirical analysis reveals that perceived organizational support significantly enhances the prohibitive voice behavior of knowledge workers—their willingness to identify organizational problems and risks to facilitate improvement (Chen et al., 2022). This logic similarly applies to teaching staff. Some scholars define teachers’ perceived organizational support as a subjective perception that arises when educators feel valued for their contributions, cared for in their well-being, and supported in their professional development, emotional needs, and work requirements by their school or educational organization (Yú et al., 2025). For instance, when schools acknowledge teachers through emotional recognition—such as respect, care, and belonging (Liu C. et al., 2023; Liu Y. et al., 2023)—provide adequate work support (Oubibi et al., 2022), and offer value affirmation through evaluation, promotion, and recognition systems (Chen et al., 2022; Pfister and Paljevic, 2024). In Yan et al. (2025) analyzed perceived organizational support (POS) and teachers’ voice behavior within a chain mediation model. They concluded that when teachers feel valued and supported by the organization, gaining psychological security and recognition, their voice behavior increases, thereby enhancing commitment. Thus, psychological capital is an indispensable factor when discussing teachers’ organizational support perception and voice behavior.

Building upon existing research, scholars further indicate that perceived organizational support not only directly encourages teachers to express constructive opinions but also indirectly enhances their willingness to offer suggestions through psychological capital. Specifically, when teachers feel valued and cared for by their organization, they are more likely to develop higher levels of psychological capital (PsyCap), including hope, optimism, resilience, and self-efficacy (Luthans et al., 2007). Even when confronted with hierarchical school structures or risks of negative evaluations, psychological capital—as a positive psychological state—can sustain proactive attitudes and generate proactive voice behavior (Wang, 2024). Furthermore, within teaching populations, psychological capital has been demonstrated to significantly enhance job satisfaction and work-life quality, serving to buffer stress and stimulate positive behaviors (Erden, 2025), thereby providing a foundation for voice behavior. However, existing evidence predominantly originates from corporate and higher education contexts, with limited research addressing kindergarten teachers who endure high emotional labor and resource constraints. Therefore, further examining the mediating role of psychological capital among kindergarten teachers not only addresses existing research gaps but also helps uncover unique patterns of voice behavior among early childhood educators within the preschool setting.

Kindergarten teachers significantly impact not only their own development and that of their peers but also profoundly influence the physical and mental health of preschool children. Currently, within the Chinese educational context, research on kindergarten teachers primarily focuses on their professional development (Liu et al., 2022), teacher-child interactions (Liu et al., 2022), and reforms in early childhood teacher education (Suo et al., 2024). Research on kindergarten teachers’ behavioral patterns is extremely scarce, including a lack of focus on VB (Qu, 2024). This stems from several reasons: firstly, in traditional educational concepts, the teacher’s role is often seen more as an executor rather than an active voice (Hao, 2011); secondly, the introduction and localization of VB theory within China’s preschool education field requires further strengthening (Duan, 2011); thirdly, compared to universities and primary/secondary schools, societal emphasis on early childhood education is insufficient (Yang, 2018). However, to cope with an increasingly dynamic and unpredictable social environment (Bennett and Lemoine, 2014), relying solely on leaders to perceive the organizational environment is insufficient to sustain organizational development. Consequently, organizational structures are transforming toward greater flatness and flexibility, making employees’ active participation and voice increasingly crucial for promoting enterprise development (Morrison, 2011; Grant, 2013).

Furthermore, within China’s specific context, establishment status is regarded as an identity marker (Wang and Wu, 2018). Establishment status means enjoying a series of benefits such as lifelong employment, stable income, generous labor protection and welfare, lifelong social security, preferential housing and material supply, and preferential treatment for children’s education (Shang et al., 2014). In contrast, employees without establishment status cannot enjoy institutional privileges, have poorer job stability, lack financial security, and also face differences in social status (Liu et al., 2021). At the same time, this “establishment consciousness,” combined with the welfare system, fosters strong identification with the work unit, influencing job stability and employees’ sense of belonging. Research on educational institutions has found that non-formal employees (without establishment status) exhibit fewer organizational behaviors compared to formal employees (with establishment status) and are less likely to engage in VB that might affect their own interests, which is highly detrimental to organizational management (Kong, 2017). Compared to teachers in primary, secondary, and higher education institutions, kindergarten teachers exhibit significantly lower staffing coverage rates, with a large number operating outside formal staffing quotas. This disparity in employment status directly impacts teachers’ compensation security, professional identity, and psychological safety (He et al., 2023), subsequently influencing their perceived organizational support and willingness to voice suggestions (Di et al., 2023). Therefore, establishment status among kindergarten teachers is not merely an institutional identity variable but also a key moderating factor determining their occupational stability and willingness to speak up.

However, current research on the role of dynamic individual psychological factors in promoting VB is still limited. Studies exploring the interrelationships between organizational and individual factors and their mechanisms influencing employee VB are also scarce. Therefore, this study examines the relationship between perceived organizational support and kindergarten teachers’ voice behavior, using psychological capital as a mediating factor and establishment status as a moderating variable.

## 2 Literature review

### 2.1 Theoretical foundation

Social exchange theory posits that interpersonal relationships are formed and maintained through reciprocal norms, wherein the recipient of benefits feels obligated to reciprocate (Blau, 1964). Within organizational contexts, employees who perceive organizational support develop a sense of obligation to repay the organization, manifesting through positive attitudes and behaviors (Eisenberger et al., 1986). Previous research also indicates that in educational settings, teachers’ perceived organizational support enhances their professional commitment and fosters additional role behaviors (Oubibi et al., 2022). Thus, this reciprocal mechanism similarly applies to examining the direct relationship between kindergarten teachers’ perceived organizational support (POS) and their voice behavior (VB).

Conservation of Resources Theory (COR) posits that individuals continuously strive to acquire, maintain, and accumulate resources they deem important to cope with stress and achieve goals (Hobfoll, 1989; Hobfoll et al., 2018). In work settings, perceived organizational support (POS) serves as a critical external resource that not only alleviates individual stress but also fosters the development of positive psychological resources (Ho and Chan, 2022). This internal psychological resource encompasses positive traits such as hope, optimism, resilience, and self-efficacy, helping individuals maintain a positive state when facing complex tasks or organizational stress (Wang, 2024), thereby influencing the emergence of voice behavior (Cooper et al., 2025). For kindergarten teachers, their work is characterized by emotional exhaustion and tedious tasks, making them highly susceptible to resource depletion (e.g., professional burnout). In this context, perceived organizational support (POS) serves as a crucial external work resource that effectively buffers job stress and is converted into teachers’ internal personal resources—psychological capital (PsyCap). The abundance of this psychological resource provides educators with sufficient “capital” to engage in resource-consuming behaviors like voice behavior. Thus, this logical pathway provides a robust theoretical basis for understanding why kindergarten teachers can actively voice concerns within organizational contexts.

The Job Demands-Resources Model (Bakker and Demerouti, 2007) offers a dynamic framework explaining how job characteristics influence employee performance. This theory posits that the utility of job resources is moderated by other contextual factors. Since voice behavior is inherently an off-role behavior carrying potential risks—such as negative evaluations or even career jeopardy (Um-e-Rubbab and Naqvi, 2020)—employees must weigh its benefits (rewarding the organization, improving conditions) against its costs (interpersonal conflict, negative evaluations from superiors, or career risks) before engaging in such behavior (Ayop and Ishak, 2023; Luo et al., 2024). Within the specific context of China’s preschool education system, establishment status—as a symbol of long-term contractual security—serves as an institutional safeguard provided by the organization. Establishment status provides teachers with a core professional resource, ensuring occupational security and job stability, which significantly mitigates the potential risk costs associated with voice behavior (e.g., marginalization or dismissal) (He et al., 2023; Liu et al., 2021). Thus, establishment status fully unleashes the motivational effect of POS, offering a robust and profound theoretical explanation for the emergence of voice behavior.

### 2.2 Relationship between perceived organizational support and voice behavior

Extensive empirical research across diverse industries and groups confirms that perceived organizational support (POS) serves as a key antecedent variable for stimulating employee voice behavior (Nazeer and Khan, 2025; Glauser, 1984; Milliken et al., 2003; Li et al., 2012; Yang, 2018). This robust relationship has also been validated in studies focusing on teaching populations. Findings from a Chinese university sample indicate that POS significantly and positively predicts inhibitory voice behavior (Yang, 2018). This robust relationship has also been validated in studies targeting the teaching population. Findings from a Chinese university sample indicate that POS significantly and positively predicts inhibitory voice behavior, and this relationship is moderated by organizational digitalization levels, suggesting that POS can effectively promote teachers’ voice behavior in educational contexts as well (Chen et al., 2022). Simultaneously, multiple studies have confirmed that leadership support factors enhance teachers’ voice behavior. For instance, inclusive leadership fosters both facilitative and inhibitory advice among university faculty by creating a supportive atmosphere (Liu C. et al., 2023; Liu Y. et al., 2023). Distributed leadership operates through mechanisms such as “threshold-free advice-seeking” with teachers and teachers’ willingness to “go beyond their own responsibilities” in decision-making or work (De Jong et al., 2023). Research in basic education has also found that POS can enhance elementary teachers’ collective efficacy, thereby increasing their job satisfaction and positive attitudes (Zhu et al., 2024). Similarly, a Taiwanese study indicates that teachers’ willingness to offer suggestions is influenced not only by personal motivation but also by whether principals provide a supportive environment. When teachers perceive leadership as valuing and responding to their input, their willingness to speak up increases (Hsieh et al., 2024). This finding further demonstrates that organizational support not only improves teachers’ psychological well-being and organizational identification but also creates a favorable psychological and organizational environment for their positive behaviors, including voice behavior.

Focusing specifically on kindergarten teachers, their work involves high emotional demands, dual responsibilities of care and instruction, and complex home-school communication—unique characteristics that make organizational support particularly crucial. This exchange relationship manifests as kindergartens (through principals and administrators) providing teachers with job support (e.g., competitive compensation), value recognition (e.g., training and promotion opportunities), and emotional recognition (e.g., respect and care)—collectively termed ‘perceived organizational support’ (Li and Hashim, 2025; Liu C. et al., 2023; Liu Y. et al., 2023). In return, kindergarten teachers, driven by trust and a sense of obligation, are more willing to engage in risky role-transcending behaviors, such as offering constructive suggestions on curriculum design, home-school collaboration, or child assessment (i.e., ‘voice behavior’) (Chen et al., 2022). Research on kindergarten principals indicates that teachers’ perceived organizational support and voice behavior play a chain-mediated role in ethical leadership and work engagement (Yan et al., 2025).

In summary, both broad organizational behavior research and empirical evidence focused on teaching populations support the positive influence of perceived organizational support on voice behavior. Therefore, this study proposes the following hypothesis H1:

*H1*: Perceived organizational support positively effects voice behavior among kindergarten teachers.

### 2.3 The mediating role of psychological capital

Perceived Organizational Support (POS) serves as a vital source for teachers to access external resources and emotional support, making it a key factor in cultivating their psychological capital (PsyCap). Psychological capital, as a positive psychological state, encompasses self-efficacy (Lopez-Garrido, 2025; Bandura, 1977), hope (Luthans et al., 2008; Snyder, 1994), optimism (Luthans et al., 2008; Scheier and Carver, 1985), and resilience (Abbas and Raja, 2015; Luthans et al., 2008). Its development is significantly influenced by organizational support (Sihag and Sarikwal, 2015). Research indicates that within the Chinese educational context, POS significantly predicts teachers’ psychological well-being and work engagement (Wang, 2024) and enhances teacher performance by boosting self-efficacy and other means (Li et al., 2025). This demonstrates that POS serves as a crucial external driver of teachers’ psychological capital.

On the other hand, since voice behavior (VB) is inherently challenging and risky (Chou and Barron, 2016; Morrison, 2014), it requires sufficient psychological resources for support. Psychological capital serves as the core of such resources, promoting voice behavior by enhancing psychological safety (Detert and Burris, 2007; Svendsen and Joensson, 2016). Based on the Ability-Motivation-Opportunity (AMO) theory, PsyCap positively predicts both employees’ facilitative and prohibitive voice behaviors (Norman et al., 2010; Wang, 2024; Shen, 2023), and generally predicts positive voluntary behaviors (Wu and Chen, 2018). Specific studies in education further validate this relationship: psychological capital enhances teachers’ psychological empowerment and organizational identification, thereby promoting both facilitative and inhibitory voice behavior (Liu C. et al., 2023; Liu Y. et al., 2023; Alazmi, 2024). When teachers possess higher positive psychological traits, they are more inclined to proactively voice opinions (Hsieh et al., 2024). Furthermore, psychological capital is closely associated with their work-life quality and positive role-extrasystemic voice behavior (e.g., speaking up) (Erden, 2025).

In summary, this study proposes that teachers’ psychological capital serves as a key mediating mechanism linking their perceived organizational support from schools to subsequent voice behavior, based on Resource Conservation Theory and Social Exchange Theory. Teachers’ work involves significant emotional exhaustion and uncertainty, and organizational support from schools functions as an external resource (emotional identification, value identification, work support, etc.) (Eisenberger et al., 1986; Zhong, 2024), serving both as institutional safeguards and emotional resources (Liu C. et al., 2023; Liu Y. et al., 2023). Therefore, drawing on Social Exchange Theory, Conservation of Resources Theory, and the Job Demands-Resources Model, we conclude that when teachers experience strong school support, this positive experience transforms into critical personal psychological resources—enhancing their psychological capital. This manifests as efficacy in teaching and nurturing students, optimism in facing challenges of student growth, resilience in overcoming work pressures, and hope for achieving educational goals (Zhong, 2024). Abundant psychological capital makes teachers more willing to go beyond routine teaching duties. This stems from their heightened psychological security and resource reserves, enabling them to bear the risks associated with offering suggestions while firmly believing their input contributes value to school development (Qu, 2024). Therefore, school support does not directly cause teacher suggestions but indirectly encourages constructive input by nurturing their intrinsic motivation through psychological capital. Based on these academic insights, this study proposes the following hypothesis H2:

*H2*: Psychological capital mediates the relationship between teachers' perceived organizational support and voice behavior.

### 2.4 The moderating effect of establishment status on the relationship between perceived organizational support and psychological capital

In China, the teacher establishment system refers to the state’s formal recognition and allocation of teaching positions within the education system. It encompasses aspects such as job placement, responsibility assignment, personnel allocation, and corresponding compensation and benefits. The sophistication of the teacher establishment system profoundly impacts not only the stability of the teaching workforce and individual teachers’ sense of fulfillment but also directly influences the attractiveness and social standing of the teaching profession (Howard and Donnelly, 1986; Ning, 2024). In recent years, with the persistent decline in birth rates and ongoing adjustments in the regional distribution of school-age populations, the imbalance between teacher job requirements and actual staffing allocations has become increasingly severe (Wang et al., 2024). This has led to a significant decline in job security for kindergarten teachers without establishment status (Zhou et al., 2024). Amid ongoing shifts in teacher workforce size and structure, discussions surrounding teacher establishment theories, policies, and practices have intensified (Ning, 2024).

Research indicates that teachers with establishment status demonstrate significantly higher work performance, compensation satisfaction, and organizational identification compared to their non-status counterparts (Liu et al., 2021). In other words, based on the impact of establishment status on perceived organizational support—such as job stability and salary benefits—and the strong influence of identity consciousness in Chinese culture, establishment status affects kindergarten teachers’ psychological capital (Liu et al., 2021; Kong, 2017). Psychological capital, in turn, is closely correlated with work attitudes and behaviors. For instance, research indicates that highly resilient employees adopt proactive attitudes toward change and seize new development opportunities; employees with high efficacy typically choose to proactively report risks and offer constructive suggestions (Cameron and McNaughtan, 2014; Luthans et al., 2007), while those lacking these traits gradually become marginalized. Therefore, based on the existing research and theoretical framework, this study proposes hypothesis H3:

*H3*: The establishment status moderates the relationship between teachers' perceived organizational support and psychological capital.

## 3 Methods

### 3.1 Participants

Using the Questionnaire Star tool, questionnaires were distributed to kindergarten teachers in Southwest China. A total of 353 questionnaires were collected. After excluding invalid questionnaires (e.g., patterned responses, obvious contradictions between positively and negatively worded items, excessively short completion times), 314 valid questionnaires were obtained, yielding an effective response rate of 91.5%.

Basic information of the participants is as follows: 293 female teachers (93.3%). This is because most of the teachers in kindergartens are female, and there are fewer male teachers; 188 teachers from public kindergartens (59.9%); 99 teachers with establishment (31.5%). To assess the representativeness of the sample, we calculated the standardized difference (SDiff/SMD) between the sample and the population on key demographic variables by comparing the basic information in the “Number of Educational Personnel in Kindergarten” (Wen et al., 2009) published by the Ministry of Education of the People’s Republic of China (2025) and the 2024 National Education Development Statistical Bulletin (Ministry of Education of the People’s Republic of China, 2025). Results indicate minimal differences in gender (∣SDiff∣ = 0.024), public school affiliation (∣SDiff∣ = 0.029), college education or higher (∣SDiff∣ = 0.056), and establishment status (∣SDiff∣ = 0.246). Thus, the sample closely matched the population in terms of gender, public school proportion, and educational attainment structure, with standardized differences all below 0.10, indicating good representativeness. However, the standardized difference for formal employment status was 0.246, exceeding the commonly accepted threshold of 0.20. It should be noted that kindergarten teacher staffing rates exhibit significant regional variation: different provinces and municipalities show marked differences in implementing the “staffing quota” policy. In some regions, nearly all public kindergarten teachers are included in the staffing quota, while others still primarily rely on contract-based employment. Furthermore, statistical definitions of “staffed” personnel vary across different years and regions in education statistics. Therefore, while the staffing ratio in this study’s sample exceeds the national average, it remains reasonably justified. This particularity will be considered when interpreting findings, and staffing status will be incorporated into the model as a key moderating variable.

### 3.2 Measures

#### 3.2.1 Perceived organizational support (POS)

Measured using the Perceived Organizational Support Scale developed by Eisenberger et al. (1986). This version, derived from the original 36-item scale, extracts 6 high-loading items summarized into three factors: Work Support, Emotional Affirmation, and Value Affirmation (6 items total). Among them, there is one reverse-scored item in the emotional identification dimension. Previous research provides evidence for this scale (e.g.,^9008,9007^; Shore and Wayne, 1993). A 5-point Likert scale was used (1 = “Strongly Disagree,” 5 = “Strongly Agree”). The overall Cronbach’s *α* coefficient was 0.817, with coefficients for each dimension ranging from 0.747 to 0.773. KMO = 0.777, indicating acceptable reliability and validity.

#### 3.2.2 Psychological capital (PsyCap)

The psychological capital questionnaire, developed by Luthans et al. (2007), was initially revised for the Chinese version by Wen et al. (2009). It comprises 16 items, with 4 items per dimension. A 5-point Likert scale was used (1 = “Strongly Disagree,” 5 = “Strongly Agree”). The overall Cronbach’s α coefficient was 0.897, with coefficients for each dimension ranging from 0.775 to 0.826. KMO = 0.912, indicating good reliability and validity.

#### 3.2.3 Voice behavior (VB)

Measured using the Voice Behavior Scale developed by Liang et al. (2012), including two dimensions: Promotive Voice and Prohibitive Voice, with 5 items each. Respondents rated their feelings, reactions, and level of agreement using a 5-point scale (1 = “Strongly Disagree,” 5 = “Strongly Agree”). The overall Cronbach’s α coefficient was 0.915, with coefficients for each dimension being 0.873 and 0.891. KMO = 0.933, indicating good reliability and validity.

#### 3.2.4 Establishment status (ES)

This study treats teachers’ establishment status as a moderator variable. This variable is an objective dichotomous variable rather than a latent construct measured via a scale. In the demographic section of the questionnaire, teachers were asked to report whether they held formal establishment. Data were coded as “0 = establishment, 1 = non-establishment.” Based on prior research (He et al., 2023; Li et al., 2025; Liu et al., 2021), establishment status frequently functions as a boundary condition influencing the relationship between organizational support and individual behavior. Therefore, this study incorporates it as a moderator in the model, primarily examining its moderating effect on the direct path between perceived organizational support and voice behavior.

#### 3.2.5 Research procedure and data processing

Data were collected via anonymous online surveys. Before administration, participants were assured of anonymity and encouraged to respond truthfully. Completion time was 5–10 min. Amos 27.0 and SPSS 27.0 were used to test the mediating and moderating effects, respectively. Specific technical methods include descriptive analysis, correlation analysis, regression analysis, and others.

## 4 Results

### 4.1 Control and test of common method bias

As this study required kindergarten teachers to simultaneously complete the POS, PsyCap, and VB scales relying on self-report data, common method bias was a potential concern. Harman’s single-factor test was applied to the 32 scale items. Seven factors had eigenvalues greater than 1, with the first factor explaining 26.815% of the variance, below the critical threshold of 40%. This indicates that common method bias was not significant. Prior to formal analysis, we tested whether the three main constructs and their respective measurement indicators converged on their corresponding factors and could be effectively distinguished. The results are shown in Table 1.

**Table 1:** Model fit indices.

Index	χ^2^/df	GFI	NFI	RFI	IFI	TLI	CFI	RMSEA
M	1.691	0.972	0.955	0.933	0.981	0.971	0.981	0.047

The IFI, TLI, and CFI values were all greater than 0.90, and RMSEA < 0.08, indicating a good model fit (Wen et al., 2009).

### 4.2 Means, standard deviations, and correlation matrix

Descriptive statistics and correlation analysis results (see Table 2) show that the overall levels of POS, PsyCap, and VB among Chongqing kindergarten teachers were moderate.

**Table 2:** Descriptive statistics and correlation analysis results.

Variable	*M*	SD	ES	POS	PsyCap	VB
POS	3.30	0.93	0.141^*^	1	0.247^**^	0.278^**^
PsyCap	3.17	0.82	0.207^**^	0.247^**^	1	0.347^**^
VB	3.50	0.93	0.130^*^	0.278^**^	0.347^**^	1
ES	–	–	1	0.141^*^	0.207^**^	0.130^*^

^*^p < 0.05, ^**^p < 0.01, ^***^p < 0.001.

POS was significantly positively correlated with PsyCap (*p < 0.001*) and with VB (*p < 0.001*). PsyCap was also significantly positively correlated with VB (*p < 0.001*). establishment status (ES) was significantly correlated with POS and VB (*p < 0.05*) and significantly correlated with PsyCap (*p < 0.01*).

### 4.3 Regression analysis of variable relationships in the model

The collinearity test revealed that the VIF values for each independent variable in this study ranged from 1.009 to 1.555, all below 2 and well below the warning threshold of 5. Tolerance values ranged from 0.658 to 0.991, all exceeding the critical value of 0.1, indicating no severe multicollinearity issues.

During analysis, we incorporated gender (GND), age (AGE), years of teaching age (TA), nature of the kindergarten (KN), and average income (AI) as control variables, generating two models using stepwise regression analysis. As shown in Table 3, POS significantly and positively predicted psychological capital (*B = 0.245, p < 0.001*), while ES significantly and negatively predicted psychological capital (*B = −0.427, p < 0.001*). Additionally, gender and educational background significantly predicted psychological capital, but the interaction effect between POS and ES was not significant. The overall model was significant, with R^2^ = 0.181. Model 3 provided the best explanation for the variance in the dependent variable VB. The regression equation is: VB = 2.241 + 0.194 × POS + 0.335 × PsyCap + 0.392 × GND − 0.022 × AGE − 0.112 × KN + 0.125 × TA + 0.070 × AI + 0.047 × EL + *ε*.

**Table 3:** Regression analysis results of the relationship between variables in the model.

Predictor	Model 1: PsyCap (M)			Model 2: Voice behavior (Y)		
	*B*	SE	*t*	*B*	SE	*t*
Constant	0.840^*^	0.424	1.98	2.241^***^	0.474	4.73
POS	0.245^***^	0.058	4.2	0.194^***^	0.055	3.54
PsyCap	–	–	–	0.335^***^	0.063	5.33
ES	−0.427^***^	0.115	−3.71	–	–	–
POS × ES	−0.116	0.098	−1.18	–	–	–
GND	−0.698^***^	0.176	−3.96	0.392†	0.2	1.96
AGE	0.039	0.062	0.63	−0.022	0.068	−0.32
KN	0.159	0.106	1.5	−0.112	0.099	−1.14
TA	0.07	0.062	1.14	0.125†	0.068	1.83
AI	−0.011	0.035	−0.31	0.070†	0.039	1.79
Education level	0.152^*^	0.066	2.3	0.047	0.075	0.63
*R* ^2^	0.181			0.192		
*F*	7.49^***^			9.05^***^		

†*p* < 0.1, ^*^p < 0.05, ^**^p < 0.01, ^***^p < 0.001.

### 4.4 Relationship between POS and VB: mediation model test

**Figure 1:**
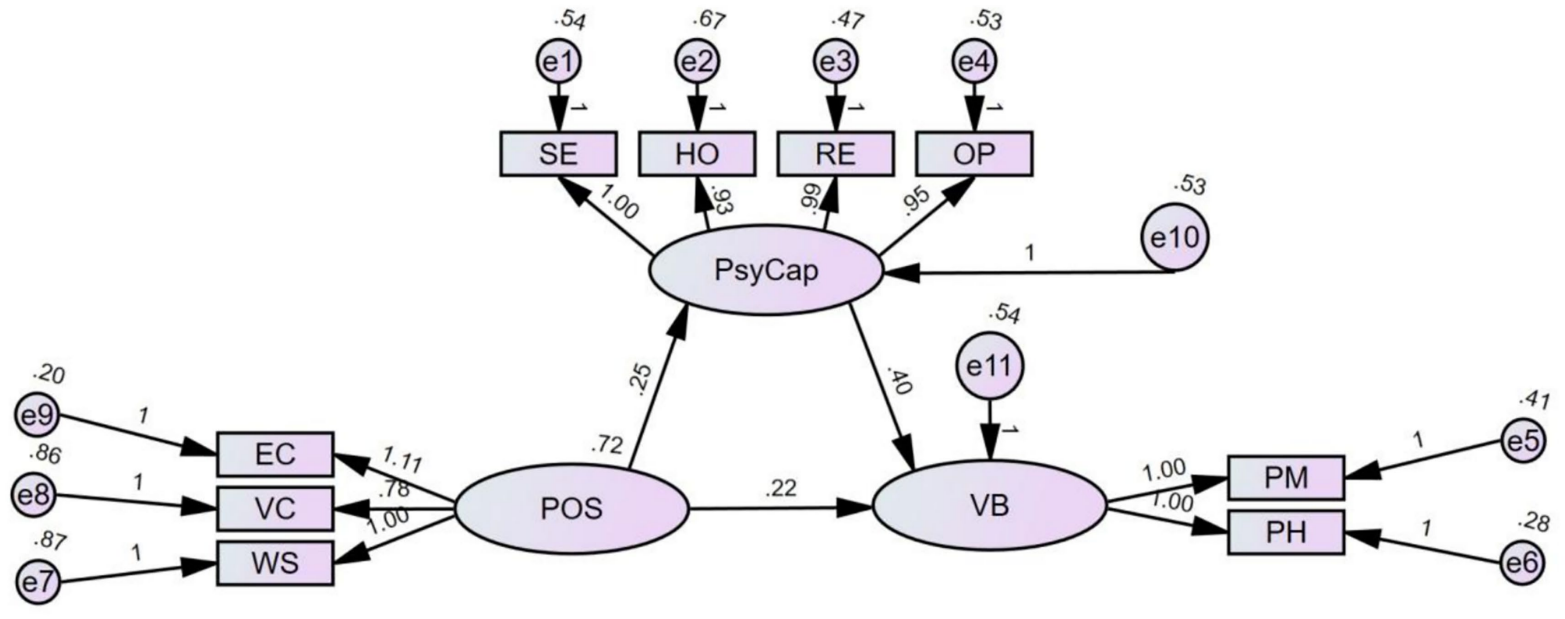
The mediating role of psychological capital between perceived organizational support and voice behavior.

To further explore the relationship between POS, PsyCap, and VB and its mechanism, structural equation modeling (SEM) using SPSS Amos 27 was employed to test the direct and indirect effects. To examine the effect of POS on kindergarten teachers’ VB, their direct relationship was first tested. After controlling for demographic variables (GND, AGE, TA, KN, AI), POS significantly predicted VB (*β = 0.216, 95% CI [0.075, 0.360], p < 0.005*), explaining 67.92% of the variance in VB. The mediating effect of PsyCap was significant (*β = 0.101, 95% CI [0.043, 0.184], p < 0.001*), explaining 31.76% of the variance in VB (Figure 1).

### 4.5 Relationship between POS and PsyCap: moderation effect test

To test the research hypotheses, this study employed the PROCESS macro (Model 7) developed by Hayes (2022) to conduct mediation analysis in SPSS 27.0. The results are presented in Table 3.

In the model with psychological capital as the dependent variable, perceived organizational support significantly and positively predicted psychological capital (*B = 0.245, t = 4.20, p < 0.001*), while establishment significantly and negatively predicted psychological capital (*B = −0.427, t = −3.71, p < 0.001*). Additionally, gender (*B = −0.698, p < 0.001*) and educational background (*B = 0.152, p < 0.05*) also exhibited significant effects. The interaction term between POS and ES was not significant (*B = −0.116, p = 0.238*), indicating that ES did not significantly moderate the predictive effect of POS on psychological capital. The overall model was significant (*F = 7.49, p < 0.001*), explaining 18.1% of the variance (Table 4).

**Table 4:** Decomposition of total, direct, and indirect effects.

Point estimate		Product of coefficients		Bootstrap 5,000 bias corrected		95%CI percentile		Percentage
		SE	*Z*	Lower	Upper	Lower	Upper	
IE	0.101	0.036	2.806	0.046	0.192	0 0.043	0.184	31.76%
DE	0.216	0.072	3.00	0.076	0.360	0.075	0.360	67.92%
TE	0.318	0.072	4.417	0.186	0 0.469	0.184	0.466	100%

In the model with voice behavior as the dependent variable, both perceived organizational support (*B = 0.194, t = 3.54, p < 0.001*) and psychological capital (*B = 0.335, t = 5.33, p < 0.001*) significantly and positively predicted voice behavior. Gender showed a marginally significant effect (*B = 0.392, p = 0.051*), while teaching experience (*B = 0.125, p < 0.10*) and AI usage level (*B = 0.070, p < 0.10*) also exerted marginally significant influences on voice behavior. The overall model was significant (*F = 9.05, p < 0.001*), explaining 19.2% of the variance.

In summary, regression results indicate that POS significantly predicts psychological capital and voice behavior; psychological capital exerts a significant positive effect on voice behavior, while establishment status does not significantly moderate the predictive role of perceived organizational support on kindergarten teachers’ psychological capital.

The Bootstrap analysis results from PROCESS Model 7 (see Table 5) indicate that, in the absence of establishment status, the indirect effect of perceived organizational support (POS) on voice behavior (VB) through psychological capital (PsyCap) is significant (Effect = 0.082, 95% CI [0.035, 0.146]), suggesting that psychological capital mediates this relationship. However, when possessing establishment status, this indirect effect weakened and became non-significant (Effect = 0.043, 95% CI [−0.003, 0.086]), including zero, indicating that the indirect path mediation effect of establishment status was not significant.

**Table 5:** Indirect effects estimated using the bootstrap method.

ES	*β*	*SE*	*95%CI* percentile	
No	0.0820	0.0283	Lower	Upper
			0.0346	0.1460
Yes	0.0431	0.0226	−0.0034	0.0862

**Figure 2:**
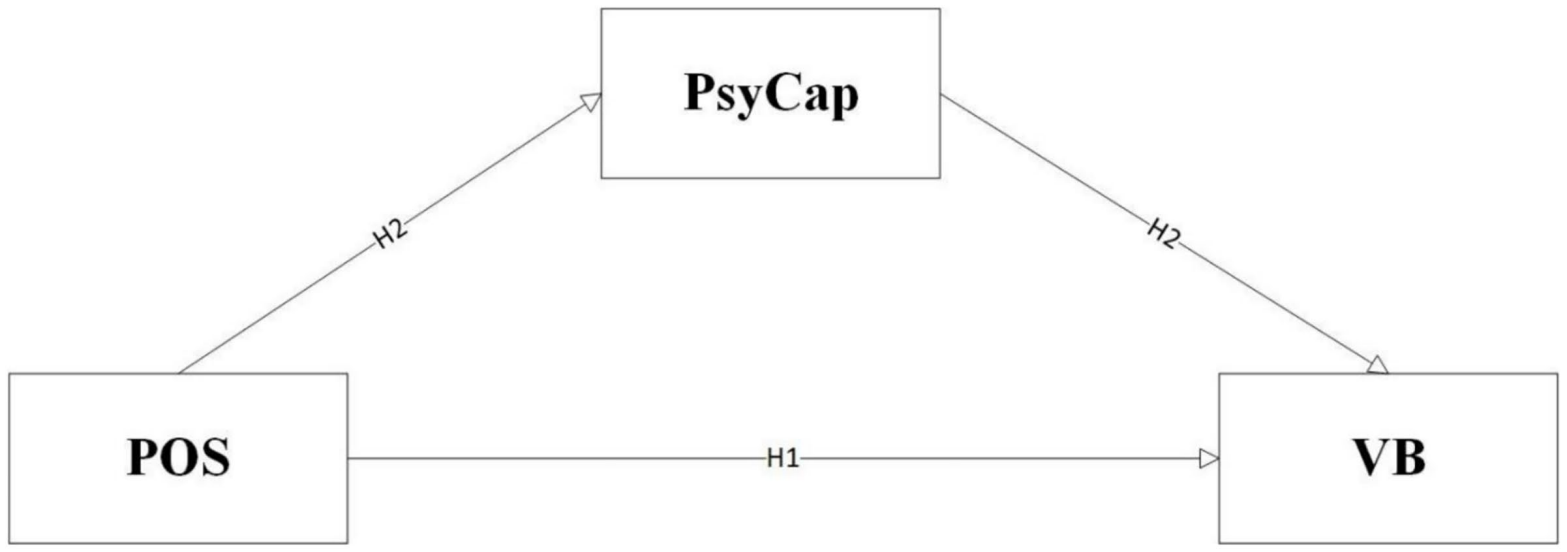
Research framework.

Thus, although this study initially constructed a mediated model with moderation based on theoretical derivation (as shown in Figure 2) and proposed Hypothesis H3: that establishment status moderates the mediating path of psychological capital, empirical analysis revealed that the moderating effect of establishment status did not reach statistical significance. Therefore, to more accurately reflect the final model validated by data analysis, we revised the framework diagram (as shown in Figure 2).

## 5 Discussion

Building on previous research and from the perspectives of organizational support theory and reciprocity theory, this study constructed a moderated mediation model with PsyCap as the mediator and establishment status as the moderator. It not only clarified the impact of POS on kindergarten teachers’ VB but also explored its mechanism (the mediating role of PsyCap) and addressed whether the establishment status of kindergarten teachers could mediate the effect of kindergarten teachers’ perceived organizational support on their voice behavior. The findings hold certain theoretical and practical significance for promoting kindergarten POS, enhancing teachers’ PsyCap, and supporting and encouraging VB among kindergarten teachers.

### 5.1 POS positively predicts kindergarten teachers’ VB

The results support Hypothesis H1: POS significantly positively predicts kindergarten the findings validate Research Hypothesis H1: perceived organizational support significantly and positively predicts kindergarten teachers’ voice behavior. This aligns with international research by scholars (Chen et al., 2022; Milliken et al., 2003; Morrison, 2014; Yan et al., 2025; Wang and Niu, 2018). Within the educational field, research has also revealed that teachers’ perceived organizational support not only enhances their organizational commitment but also significantly increases their willingness to proactively offer suggestions. Specifically, when teachers perceive support, trust, and recognition from their school, employees become motivated, leading to greater diligence and effort in their work (Hsieh et al., 2024; Liu C. et al., 2023; Liu Y. et al., 2023). In studies like Liang et al. (2012), based on China’s specific context, researchers proposed that facilitative suggestions are more readily accepted by superiors, whereas prohibitive voice may provoke disputes or conflicts. Therefore, for kindergartens, the key challenge lies in reducing kindergarten teachers’ reservations when offering suggestions and encouraging them to actively voice opinions, particularly prohibitive ones (Chen et al., 2022). Eisenberger et al. (1986) and Wayne et al. (2002) approached this from social exchange theory, noting that when employees perceive a sense of perceived organizational support (e.g., receiving valuable resources), they develop a sense of obligation and belonging, translating these feelings into positive work behaviors toward the organization. For instance, when kindergartens provide favorable working conditions (such as reasonable compensation, promotion opportunities, fair and transparent performance evaluation mechanisms, and diverse work formats), an open-minded leadership style (Detert and Burris, 2007; Yú et al., 2025; Liu et al., 2016), and a secure organizational atmosphere, employees tend to work harder, proactively assist colleagues, actively identify and resolve problems, and may even challenge existing organizational practices (Liang et al., 2012; Qian et al., 2007). This fosters greater willingness among teachers to take responsibility and offer constructive suggestions, thereby promoting the synergistic development of kindergarten educational quality and management standards.

### 5.2 Psychological capital of kindergarten teachers partially mediates the relationship between POS and VB

The mediation results confirmed research hypothesis H2: teachers’ psychological capital partially mediated the relationship between perceived organizational support and voice behavior. This aligns with findings from numerous international studies (Detert and Burris, 2007; Luthans et al., 2008; Norman et al., 2010; Svendsen and Joensson, 2016). Perceived organizational support directly and positively predicts kindergarten teachers’ psychological capital levels, and there is also a positive correlation between teachers’ psychological capital levels and their voice behavior (Yú et al., 2025; Zhong, 2024). According to self-determination theory, the fulfillment of employee needs effectively promotes work engagement, and psychological empowerment is a key manifestation of this need satisfaction. Therefore, organizational support from kindergartens, as an external resource, does not directly influence teacher work engagement but operates through employees’ psychological perceptions and evaluations. Different psychological states of teachers lead to varying interpretations of perceived organizational support, which in turn affect their behavioral outcomes (Zhong, 2024; Huang, 2010). This positive psychological capital not only helps teachers overcome psychological barriers when voicing differing opinions but also enhances their confidence and willingness to express authentic perspectives and improvement suggestions within the organization, thereby increasing the frequency of voice behavior.

### 5.3 The moderating effect of establishment status on the relationship between perceived organizational support and psychological capital

Based on the job demands-resources model, this study constructed a moderated mediation model to examine the influence of establishment status on the pathway from “perceived organizational support—psychological capital—voice behavior” among kindergarten teachers. However, results revealed that establishment status did not significantly moderate the relationship between perceived organizational support and psychological capital. This finding diverges from previous research (Liu et al., 2021; Zhou et al., 2024). Thus, Hypothesis H3 was not supported by the data, potentially due to the following reasons.

Regarding national policies, the government is actively narrowing the gap between formal and non-formal establishments, striving to implement “equal pay for equal work” and safeguard teacher benefits to eliminate status-based inequalities. As outlined in policy documents: “Private kindergartens should reasonably determine teacher salaries by referencing local public kindergarten wage levels (Ministry of Education of the People’s Republic of China, Department of Basic Education, 2018)” and “Improve wage security mechanisms for kindergarten teachers to ensure full payment and equal pay for equal work (Ministry of Education of the People’s Republic of China, 2017).” The advancement and implementation of these policies may objectively narrow the actual gap in key material benefits like compensation and welfare between tenured and non-tenured teachers. This could potentially diminish differences in psychological security and organizational commitment stemming from status disparities. Consequently, the moderating effect of establishment status on perceived organizational support regarding psychological capital may weaken.

Second, research on the public sector has also found that employees with establishment typically enjoy greater job stability and psychological security. However, this is often accompanied by organizational inertia and risk-averse tendencies, which reduce career advancement and compensation incentives, leading to decreased employee effort (Sylvia et al., 2021; Zhang et al., 2013). Studies on teachers reveal that those in the establishment are more prone to complacency and professional burnout, exhibiting weaker self-directed development awareness (Yang, 2024; Tian, 2017).

Furthermore, this study employs a binary classification to measure establishment status. While this is the mainstream approach in the field, it may fail to fully capture internal heterogeneity, overlooking finer distinctions such as contract type and duration, benefits, and clarity of career advancement pathways. This potentially dilutes underlying moderating effects. This conclusion points to a crucial theoretical direction for future research on teacher voice behavior: moving beyond institutional identity to uncover the key psychological factors that shape teachers’ perceptions of their circumstances and their voice behavior.

## 6 Conclusions and recommendations

### 6.1 Establish effective incentive systems and foster a positive organizational climate

The research shows that perceived organizational support can significantly and positively predict kindergarten teachers’ voice behavior. Considering China’s specific context, we can establish reasonable incentive systems, such as appropriate bonuses, subsidies, insurance, and leave, which can enhance teachers’ motivation and increase their perceived organizational support. The nature of the teaching profession and the realities of China’s education system often lead to teachers working overtime and experiencing excessive fatigue. Therefore, establishing certain incentive and security systems for teachers and creating an organizational atmosphere filled with respect and care (Qian et al., 2007) is crucial. Additionally, strengthening emotional support (Suo et al., 2024) allows teachers to fully utilize their professional talents in such an environment, enhancing their alignment with the kindergarten’s values (Yú et al., 2025), thereby promoting employees’ work autonomy (Schneider et al., 2013) and increasing voice behavior.

### 6.2 Value teachers’ psychological functioning and enhance their psychological capital levels

Furthermore, Psychological Capital (PsyCap) plays a partial mediating role in the impact of Perceived Organizational Support (POS) on Voice Behavior (VB), meaning that psychological capital can strengthen the positive effect of perceived organizational support on voice behavior. The Person-Organization Fit (P-O-F) theory posits that “differences in individual attitudes and behaviors can only be explained by the interaction between the individual and the characteristics of the organizational environment.” Therefore, kindergartens first need to recognize that the appropriate application of teachers’ psychological capital relates to their emotional states (Zhong, 2024). By adopting an open, inclusive, and encouraging attitude towards teachers and providing them with ample space for self-expression and facilitating conditions, teachers gain greater self-determination, ensuring higher self-efficacy in their work. Secondly, Kurtessis et al. (2017) pointed out that the higher the alignment between organizational members and organizational goals, the stronger the consistency in values, leading employees to perceive stronger organizational support and greater stimulation of positive psychological functioning. Therefore, kindergarten organizations should emphasize their well-being goals and strive to integrate them with teachers’ concepts of happy work. This facilitates teachers in applying their knowledge, abilities, strengths, and talents, promoting the occurrence of voice behavior. Finally, it is vital to value teachers’ mental health (Fan et al., 2016) and focus, in real educational settings, on the application of teachers’ positive psychological qualities such as optimism, confidence, and resilience, and the appropriate deployment of psychological resources (Zhong, 2024).

### 6.3 The deep-seated role of establishment and management implications

Although the research findings indicate that establishment status does not significantly moderate the relationship between perceived organizational support and psychological capital, this does not imply that establishment factors lack importance. On the contrary, establishment status may have been internalized by employees as a deeper psychological cognition and value orientation, thereby subtly influencing their attitudes and behavioral performance. Therefore, in organizational management and policy formulation, it remains essential to fully recognize the profound impact stemming from establishment levels and to propose corresponding improvement measures accordingly.

Faced with China’s continuously declining birth rate, the attractiveness of preschool education majors in universities and the kindergarten teaching profession is gradually weakening. To stabilize the kindergarten teaching workforce and enhance their professional security, this study suggests, first, scientifically adjusting teacher establishment standards based on student-teacher ratios (Liu C. et al., 2023; Liu Y. et al., 2023). Secondly, improve the dynamic management mechanism for establishments to protect the rights and interests of private and non-established teachers (Ministry of Education of the People’s Republic of China, Department of Basic Education, 2018), striving to ensure that the welfare and other legitimate rights of non-established kindergarten teachers are guaranteed, thereby reducing the gap in treatment and rights between established and non-established teachers. At the government level, investment in public education funding should be increased to raise the compensation and security levels for non-established kindergarten teachers, narrowing the income gap between established and non-established teachers. At the kindergarten level, a fair competitive environment should be fostered, minimizing the influence of establishment status on teachers, and providing equal career development opportunities for non-established teachers (Zhou et al., 2024; Liu et al., 2021).

## 7 Limitations and future directions

This study focused on examining the impact of perceived organizational support on kindergarten teachers’ voice behavior, exploring the mediating role of psychological capital and the moderating role of establishment status. Although the sample was selected based on specific criteria, China’s large population suggests that increasing the sample size could enhance the generalizability of the findings. Additionally, the study found that establishment status, as an institutional variable, did not support a moderating role. Future research should further explore and deepen understanding of the underlying processes through which the establishment status exerts its effects.

## 8 Conclusion

This study reveals a mediating mechanism whereby perceived organizational support influences voice behavior through psychological capital. This core finding holds significant theoretical value, demonstrating the cross-group stability of psychological capital and providing more universal evidence for teacher organizational support theory. Although the moderating role of establishment status was not supported, this research lays a crucial foundation for future in-depth exploration of the more complex mechanisms underlying teacher voice behavior.

## Data Availability

The original contributions presented in the study are included in the article/supplementary material, further inquiries can be directed to the corresponding author/s.
